# Genome sequencing and transcript analysis of *Hemileia vastatrix* reveal expression dynamics of candidate effectors dependent on host compatibility

**DOI:** 10.1371/journal.pone.0215598

**Published:** 2019-04-18

**Authors:** Brenda Neves Porto, Eveline Teixeira Caixeta, Sandra Marisa Mathioni, Pedro Marcus Pereira Vidigal, Laércio Zambolim, Eunize Maciel Zambolim, Nicole Donofrio, Shawn W. Polson, Thiago Andrade Maia, Chuming Chen, Modupe Adetunji, Brewster Kingham, Ronaldo José Durigan Dalio, Mário Lúcio Vilela de Resende

**Affiliations:** 1 Programa de Pós-graduação em Biotecnologia Vegetal, Universidade Federal de Lavras, Lavras, Minas Gerais, Brazil; 2 Empresa Brasileira de Pesquisa Agropecuária (Embrapa-Café), Brasília, Distrito Federal, Brazil; 3 Departamento de Fitopatologia, Universidade Federal de Lavras, Lavras, Minas Gerais, Brazil; 4 Núcleo de Análises de Biomoléculas, Universidade Federal de Viçosa, Viçosa, Minas Gerais, Brazil; 5 Departamento de Fitopatologia, Universidade Federal de Viçosa, Viçosa, Minas Gerais, Brazil; 6 Department of Plant and Soil Sciences, University of Delaware, Newark, Delaware, United States of America; 7 Center for Bioinformatics and Computational Biology, Delaware Biotechnology Institute, Newark, Delaware, United States of America; 8 Sequencing and Genotyping Center, Delaware Biotechnology Institute, University of Delaware, Newark, Delaware, United States of America; 9 Instituto Agronômico de Campinas, Centro de Citricultura “Sylvio Moreira”, Cordeirópolis, São Paulo, Brazil; University of Nebraska-Lincoln, UNITED STATES

## Abstract

Coffee leaf rust caused by the fungus *Hemileia vastatrix* is one of the most important leaf diseases of coffee plantations worldwide. Current knowledge of the *H*. *vastatrix* genome is limited and only a small fraction of the total fungal secretome has been identified. In order to obtain a more comprehensive understanding of its secretome, we aimed to sequence and assemble the entire *H*. *vastatrix* genome using two next-generation sequencing platforms and a hybrid assembly strategy. This resulted in a 547 Mb genome of *H*. *vastatrix* race XXXIII (Hv33), with 13,364 predicted genes that encode 13,034 putative proteins with transcriptomic support. Based on this proteome, 615 proteins contain putative secretion peptides, and lack transmembrane domains. From this putative secretome, 111 proteins were identified as candidate effectors (EHv33) unique to *H*. *vastatrix*, and a subset consisting of 17 EHv33 genes was selected for a temporal gene expression analysis during infection. Five genes were significantly induced early during an incompatible interaction, indicating their potential role as pre-haustorial effectors possibly recognized by the resistant coffee genotype. Another nine genes were significantly induced after haustorium formation in the compatible interaction. Overall, we suggest that this fungus is able to selectively mount its survival strategy with effectors that depend on the host genotype involved in the infection process.

## Introduction

*Coffea arabica* (Arabica coffee) and *C*. *canephora* (Conilon coffee), are the two most cultivated coffee species in Brazil, which is the leading producer of coffee with approximately 43 million bags harvested in 2015 [1]. Due to the high quality of its beverage, *C*. *arabica* is the species most cultivated worldwide. However, a major drawback is that it is highly susceptible to *Hemileia vastatrix* Berkeley and Broome (1869), the coffee leaf rust pathogen. The fungus is widespread in coffee growing regions causing significant yield losses and its chemical control can lead to increased production costs. The best control strategy for the disease has been the use of resistant cultivars [2] [3], which have been developed through breeding programs in various countries. Another factor aggravating this disease is that the fungus has been able to overcome the resistance of recently released cultivars and has more than 50 physiological races described globally [3] [4]. In Brazil, previous studies differentiated 15 races named as I, II, III, VII, X, XIII, XV, XVI, XVII, XXII, XXIII, XXIV, XXV or XXXI, XXXIII, and XXXVII [3]. Race XXXIII was recently identified in Brazil [5] and overcame the resistance of cultivars Catimor, Tupi Amarelo and Catucaí Amarelo, which are derived from either Icatu or Híbrido de Timor, the major rust resistance parental donors [6] [7] [8] [3]. In this way, the presence of the rust virulent race XXXIII in the field may impose a serious threat to any coffee orchard worldwide.

The infection process of *H*. *vastatrix* on coffee leaves starts with the germination of urediniospores on the abaxial leaf surface, formation of appressoria over a stoma and subsequent penetration, spread of penetration hypha into the substomatal chamber, and further inter and intracellular colonization. At the tip of the penetration hypha, two thick lateral branches resembling an anchor are formed, a unique characteristic of *H*. *vastatrix*. Haustorial mother cells (HMC) are formed at each lateral branch of the anchor, giving rise to haustoria [9].

Fungal structures can develop at different times depending on the host genotype. In susceptible coffee cultivars, hyphae are formed from the majority of the infection sites, thus resulting in the formation of a large number of haustoria in the palisade and spongy parenchyma as well as in the upper epidermal cells. In these compatible interactions, where disease is the outcome, the infection process culminates with the colonization of mesophyll cells and a urediniosporic sporulation after 20 days post penetration [10] [4]. In resistant coffee cultivars, fungal growth is ceased usually after the formation of the first haustorium [11], and this is denoted as post-haustorial resistance. Other coffee genotypes may present pre-haustorial resistance as observed in the field and similar to non-host resistance, which prevents formation of haustoria [12] [13] [9].

Similar to other pathogenic fungi, *H*. *vastatrix* establishes its parasitic colonization by secreting effector proteins that modify the structure and function of host cells. Some of the effectors may also manipulate the plant immunity to increase pathogen fitness as observed in other plant-pathogen interactions [14] [15] [16]. R proteins encoded by resistance genes can recognize some of these effector proteins and thus trigger plant defense responses [17] [18]. The identification and characterization of these effectors, as well as their correlated R genes, are the first steps for understanding the molecular mechanisms underlying the gene-for-gene interaction and for a more focused breeding program for resistance to coffee leaf rust. Thus, it is crucial that genome and transcriptome data are available for a more thorough investigation and analysis of fungal effector proteins.

Over the past 10 years, advances in Next Generation Sequencing (NGS) technologies and decreasing sequencing costs allowed an increase in the number of sequenced genomes, especially of plant pathogenic fungi causing important diseases in major agricultural crops. To date, genomes of five important rust fungi have been published: *Melampsora larici-populina* (101.1 Mb, [19]), *Puccinia graminis* f. sp. *tritici* (88.6 Mb, [19]; [20]), *Puccinia striiformis* f. sp. *tritici* (64.8 Mb, [21]), *Melampsora lini* (189 Mb, [22]), and a partial draft genome of *H*. *vastatrix* (333 Mb, [23]). Genome-wide analyses of these rusts revealed a large catalog of secreted proteins, some of which are candidate effectors.

In general, and when compared to non-biotrophic fungi, rust fungal species have large genome sizes, including *H*. *vastatrix* among the largest genomes of rust fungi. The large size of the fungal nuclear genome was measured by flow cytometry, as ranging from 733.5 Mb [24] to 796.8 Mb [25]. However, the lack of a well-assembled genome and the unknown full repertoire of effector proteins has significantly impeded the investigation of *H*. *vastatrix* biology and biotrophic interaction with its host as well as slowing the progress of a focused breeding program for resistant cultivars.

Therefore, the main goal of this study was to deep sequence the genome of *H*. *vastatrix* urediniospores of race XXXIII using a combined sequencing strategy with long (PacBio) and short (Illumina) reads and an integrated genome assembly method for achieving a high-quality reference genome. We used *in silico* methods to predict secreted proteins, and a subset of their corresponding genes were subject to time course-based, gene expression studies. Together, our data provide the most comprehensively sequenced and assembled coffee rust genome to date and an initial description and characterization of the putative secretome.

## Material and methods

### Fungal material

The *H*. *vastatrix* monopustular isolate Hv-02, characterized as race XXXIII, was used in this work. It was massively multiplied on coffee seedlings, *Coffea arabica* cv. Caturra (CIFC 19/1), as described previously [26] to result in prolific urediniospore production. Conditions (detailed below) were created to ensure the existence of sufficient concentration of fungal suspension for successive inoculations on seedlings. The fungal suspension was obtained by adding 2 mg of urediniospores in 2.0 ml sterile microfuge tubes containing 1000 μl of sterile distilled water. The suspension was vortexed for 20 minutes and centrifuged at 2,500 × g for 10 minutes. These procedures were performed three times to ensure the removal of impurities. Spores were uniformly spread on polyethylene Petri dishes containing a thin layer of agar and water. These Petri dishes were stored at 22°C for 16 hours in the dark to allow spore germination. After germination, urediniospores were scraped from Petri dishes, frozen in liquid nitrogen, and stored at -80°C until further processing. Liquid nitrogen was used to grind the samples for DNA/RNA extraction.

### DNA and RNA extractions

Genomic DNA was extracted using the Mobio Power Microbial Maxi DNA Isolation kit (MOBIO Laboratories Inc., Carlsbad, CA, USA). After extraction, DNA concentration was measured using NanoDrop Spectrophotometer ND-2000 (Thermo Fisher Scientific Inc., Waltham, MA, USA), and its quality was determined by electrophoresis in agarose gel.

For RNA extraction, viable urediniospores were inoculated on two coffee cultivars: on the resistant coffee seedlings, Híbrido de Timor (CIFC 832/1), and on the susceptible coffee seedlings, *C*. *arabica* cv. Caturra (CIFC 19/1). Leaves were collected at 12, 24, 48 and 72 hours after inoculation and immediately frozen by immersion into liquid nitrogen. Samples were stored at -80°C until further processing. Total RNA was extracted using RNeasy Plant Mini kit (Qiagen, Hilden, Germany), according to manufacturer’s instructions.

### cDNA synthesis

The cDNA synthesis was performed using Impron II reverse transcriptase kit (Promega, Madison, WI, USA) with 1μg of total RNA added according to manufacturer’s instructions. The efficiency of the cDNA synthesis process was assessed by PCR using the following reference genes: β-tubulin and Cytochrome c oxidase subunit III. The integrity of PCR amplification products was verified by electrophoresis in 1.2% agarose gel.

### Sequencing and assembly of the genome

Genomic DNA sequencing libraries of the race XXXIII (Hv33) were sequenced using two platforms: Pacific Biosciences PacBio RS II (Pacific Biosciences, Menlo Park, CA, USA), and Illumina HiSeq 2500 (Illumina, San Diego, CA, USA). All sequencing was performed at the University of Delaware Sequencing and Genotyping Center (Delaware Biotechnology Institute, Newark, DE, USA). Approximately 10 μg of DNA from germinated urediniospores (protocol described above) were sheared to 5 kb fragments using the Covaris instrument (Covaris S2 Adaptive Focused Acoustic Disruptor with CryoPrep), and were used to build two single-end libraries, using PacBio Library Preparation kit, according to manufacturer’s instructions, and sequenced in the Pacific Biosciences RSII Single-Molecule Sequencer. The first library was sequenced using 15 Single Molecule Real Time (SMRT) cells and combinations of two chemistries, namely, XL-C2 (11 SMRT) and P4-C2 (4 SMRT). The second library was sequenced based on 17 SMRT cells by using only the P5-C3 chemistry. For Illumina sequencing, approximately 10 μg of DNA from germinated urediniospores (protocol described above and the same material used for the PacBio sequencing) was used to construct four libraries and sequenced with paired-end 151 bp reads.

FastQC (The Babraham Institute, Babraham, UK) was used to assess the quality of reads generated with the Illumina platform. CLC Genomics Server version 6.0.4 (CLC bio, Aarhus, Denmark) was used for quality trimming (q<0.001 with no ambiguous nucleotides), adapter removal, and filtering of any reads shorter than 35bp. Overlapping paired end reads were merged previous to assembly using CLC. For the long reads generated with the PacBio platform, the quality of reads were assessed and filtered using default settings of PacBio SMRT Portal version 2.2.0.p2 (SMRT Analysis).

**Fig 1:**
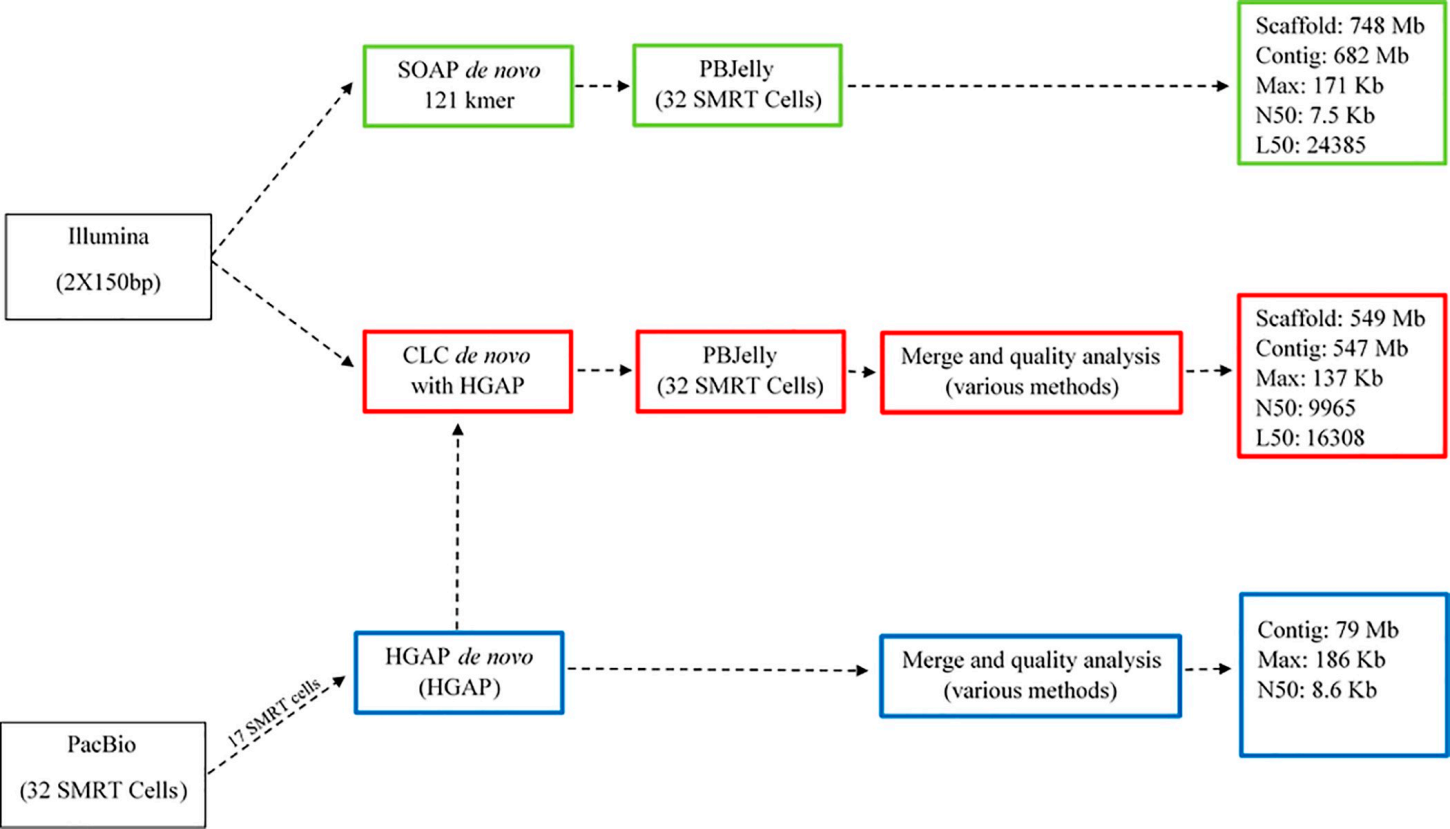
Schematic diagram describing different strategies used in the assembly the *H*. *vastatrix* genome (race XXXIII). Blue rectangles refer to the first assembly strategy, which used 17 SMRTcells with P5-C3 chemistry obtained on the PacBio RS2 platform in the HGAP assembler. Green rectangles refer to the second assembly strategy, which included a *de novo* assembly using reads obtained on the Illumina platform with SOAP *de novo* 2 assembler and subsequently improved with 32 SMRT cells of PacBio reads using PBJelly software. Red rectangles refer to the third strategy, which performed a *de novo* assembly using reads obtained from both next generation sequencing platforms with additional improvement with PBJelly software.

A hybrid genome assembly approach was used to obtain a consensus genome build: (i) assembly of the Illumina reads; (ii) assembly of the PacBio reads; and (iii) hybrid assembly approaches incorporating data from both platforms. The best of these approaches as determined by criteria including N50, longest contig/scaffolds, and read mapping accuracy criteria are summarized in Fig 1, although many additional approaches were also applied. Among the best performing approaches, high quality Illumina reads were separately de novo assembled using the CLC Genomics Workbench version 7.5 (CLC bio, Aarhus, Denmark) and the SOAP De-Novo version v2r215 [27] using kmer parameter sweeps, with the 121 bp kmer assembly from SOAP Denovo2 determined to be the best. PacBio long reads were also independently assembled using the PacBio SMRT Portal HGAP algorithm (versions 1 and 2; [28]), with version 2 performing best. The contigs from the HGAP assembly with the best assembly parameters were used as “Guide Contigs” for a new *de novo* assembly using CLC with the Illumina raw data.

The resulting set of scaffolds from both the SOAP de novo assembly and the HGAP/CLC Hybrid assembly were further joined and gaps were filled using PacBio long read data in PBJelly (v14.1.14; [29] in conjunction with the BLASR mapping tool ([30]; options: -minMatch 8 -minPctIdentity 70 -bestn 10 -nCandidates 20 -maxScore -500). Illumina raw data was mapped to the joined and filled scaffolds using CLC, and non-ambiguously mapping reads were used to correct nucleotide and InDel errors, typically in the PacBio-filled regions. Read mapping data was further utilized in CLC (Illumina reads) and PacBio SMRT Portal (PacBio reads) to identify potential mapping breakpoints and variations in genome coverage that might be indicative of mis-assembly, and manual genome finishing was performed utilizing available results. Illumina data from this study, and the 454 sequence data (NCBI SRA: SRR833265, SRR833266 –quality trimmed as previously described but to q>0.05) used to generate the previously existing genome assembly (HvCat) (NCB: GCA_003057935.1) [23], were mapped to the Hv33 assemblies. Besides, by CLC Genomics Workbench, the HvCat assembly were mapped using both the default read mapping settings (80% identity over 50% read length) and a higher stringency setting (90% identity over 90% read length). Final SOAP and CLC/HGAP assemblies were evaluated using these data, as well as annotation results from each. The CLC/HGAP assembly performed significantly better, particularly in respect to percentage of Illumina reads which could be correctly aligned, as such this assembly is the one reported herein.

### Annotation of genome and gene prediction

The structural annotation of genome and gene prediction were performed using the MAKER pipeline designed for emerging model organism genomes [31]. Our pipeline included masking of repeats using RepeatMasker (version 4.0.7; [32]) and the RepBase Puccinales library (build 1.24.19; [33]. This was followed by three iterative passes of gene calling using the Maker pipeline (version 2.31.10; [31]). First pass included all proteins from UniProt database under the order Puccinales (171,373 total proteins). The second pass combined transcripts assembled using CLC reference-guided Transcript Discovery (version 11.0.1), Trinity reference-guided assembly [34], and Trinity *de novo* assembly of RNA-Seq data from three libraries of the same race of *H*. *vastatrix* [35]. Similarly, assembled transcripts from another *H*. *vastatrix* race HvCat downloaded from NCBI SRA (SRR1124793; [23]; and 352,146 NCBI downloaded ESTs from *H*. *vastatrix* CIFC isolate 178a (ERR106426; [36]) were used. All such sequence data from *H*. *vastatrix* races other than XXXIII was treated as being data from “related organisms” by Maker. Gene models from prior Maker iterations were used to train SNAP (GSL Biotech, Chicago, IL, USA) and Augustus (version 3.22; [37]) models, which were used along with a custom-trained GeneMark ES (version 2.5p; [38]) for *ab initio* gene calling in concert with the protein/transcript gene mapping data in Maker iterations 2 and 3. BUSCO (version 3.0.2; [39]) and CEGMA (version 2.5; [40]) were used to assess completeness of assembly/annotation with comparison to other genome assemblies from the order Puccinales–HvCat: *H*. *vastatrix* isolate HvCat (GCA_003057935.1), Mp: *Melampsora larici-populina* 98AG31 (GCF_000204055.1); Ml: *Melampsora lini* CH5 (JGI Genome portal: https://genome.jgi.doe.gov/Melli1/Melli1.info.html); Pg: *Puccinia graminis* f. sp. *tritici* CRL 75-36-700-3 (GCF_000149925.1); Ps: *Puccinia striiformis* f. sp. *tritici* (GCA_001936605.2) [19, 22]. BUSCO was run in both genome and protein modes and using the basidiomycota_odb9 conserved genes library.

### Annotation of proteome

Closest known homologs for the predicted protein coding genes with transcriptome support from the *H*. *vastatrix* genome (Hv33) were determined using BLAST version 2.6.0 [41] to search the GenBank non-redundant database (GenBank nr) and UniProt Knowledgebase (UniProtKb) using BLASTp (e-value < 10^−5^). The proteins were also aligned to the EuKaryotic Orthologous Groups (KOG) using Reverse Position-Specific BLAST (RPS-BLAST). The proteins were further annotated with gene ontology (GO) terms using Blast2GO [42].

### Secretome analysis

The secretome of *H*. *vastatrix* was predicted using a pipeline combining different bioinformatics approaches. The proteins containing signal peptides were selected by SignalP version 4.0 [43] (cutoff D-Score = 0.45). The proteins flagged as related to secretory pathway signal peptide (SP) were selected by TargetP version 1.0 [44]. In addition, Wolf Psort [45] was also used to select proteins based on subcellular localization prediction. Then, the proteins without transmembrane domains were selected by TMHMM version 2.0 [46]. Following this, residues of cysteine and motifs characteristic of effectors, such as [YWF]xC, CxxC and CxxxC, were searched for in each secreted protein identified.

We predicted the secretome of four rust species (*P*. *graminis* f. sp. *tritici*, *P*. *striiformis* f. sp. *tritici*, *M*. *larici-populina* and *M*. *lini*) using the same pipeline that we used for predicting the secretome of *H*. *vastatrix*, and thus aiming an unbiased comparison. Then, the predicted secretomes were clustered based on sequence similarities using OrthoVenn [47] (e-value: 10^−5^; inflation value: 1.5).

Sequences of proteins of *H*. *vastatrix*, which did not have a hit, were compared with secreted proteins described in [36, 48] using the search tools BLASTp (*e*-value = 10^−5^) and BLASTx (*e*-value = 10^−10^), as described above. Those sequences were compared with RNA-Seq data described in [35], which were obtained by sequencing libraries from the *H*. *vastatrix*-coffee leaves interaction. These libraries were obtained using *C*. *arabica* cv. Caturra (CIFC 19/1) and Híbrido de Timor (CIFC 832/1) inoculated using fresh spores of *H*. *vastatrix* race XXXIII, seeking to establish compatible and incompatible interactions, respectively. In this study, only libraries obtained from incompatible interaction in two inoculation periods, i.e., 12 and 24 hours after inoculation were used.

### Primer design

Primer Express 2.0 software (Applied Biosystem, Carlsbad, CA, USA) was used to design specific primers for genes that encode *H*. *vastatrix* candidate effectors (S1 Table).

The beta-tubulin (*β-tubulin*), cytochrome C oxidase subunit III (*CytIII*,), 40S ribosomal protein, and glyceraldehyde 3-phosphate dehydrogenase (*GAPDH*) genes, previously used for expression analysis in *H*. *vastatrix* [49], were tested by RT-PCR as endogenous reference genes. The RT-PCR was carried out as an initial measure of amplified products using cDNA synthesized from RNA of pathogen samples, cDNA synthesized from RNA of coffee leaf samples only, and negative control consisting of nuclease-free water. The primers that showed amplification only for the cDNA from the pathogen were validated and used as endogenous reference genes.

Each 20 μL RT-PCR reaction volume included the following at final concentrations of: 1×buffer, 1.0 mM MgCl_2_, 60 μM dNTP, 0.1 μM of each forward and reverse primers, 1 U Taq polymerase and 50 ng of cDNA sample. The PCR amplified products were separated by electrophoresis in 1.2% agarose gel.

### Real-Time qPCR

Real-Time quantitative Polymerase Chain Reaction (RT-qPCR) was carried out in the ABI PRISM 7500 Real Time PCR Systems (Applied Biosystems, Carlsbad, CA, USA). The SYBR Green system (Thermo Fisher Inc., Waltham, MA, USA) was used to detect PCR amplified products. Each 10 μl RT-qPCR reaction volume contained 50 ng cDNA, 0.4 μM of each forward and reverse primers, 5 μl (50% v/v) of Power SYBR Green PCR Master Mix solution, and 2.2 μl of nuclease-free water. The thermal cycling conditions consisted of initial denaturation step at 95°C for 10 minutes, followed by 40 cycles at 94°C for 15 second and 60°C for 1 minute. At the end of each reaction, a dissociation curve analysis was performed by heating the amplicon from 60 to 95°C in order to confirm the specificity of the amplification. Standard curves using five points of cDNA serial dilution were used to optimize the concentration of cDNA and primer efficiencies. For each dilution point, cycle threshold value (Ct) efficiency was estimated for each primer, including target genes and selected reference genes.

Three technical replicates for each of three existing biological replications for each sample (resistant plants and susceptible plants) were performed at the following four time-points: 12, 24, 48, and 72 hours after inoculation (hai). The data were analyzed with REST software proposed by [50].

## Results

### Sequencing and assembly of *Hemileia vastatrix* genome

Fresh urediniospores of *H*. *vastatrix* race XXXIII incubated on polystyrene plates for 16 hours at 22°C in the dark (as described in Methods) resulted in approximately 80% germination. The sequencing of long read libraries rendered approximately 8.39 Gbp of data from 32 SMRT cells on the PacBio RS II platform. Following quality screening, 5.73 Gbp comprised of 920,326 long-reads were obtained. The N50 of PacBio reads was estimated at 8.6 kb with an expected genome coverage >20X. The Illumina HiSeq platform produced 264,721,502 short-reads. These reads totaled >100X coverage for the expected genome size of *H*. *vastatrix* (~733.5 Mb according to [24]).

Multiple strategies were tested for the hybrid genome assembly for the dual-platform sequencing, the most successful strategies are outlined in Fig 1. Our approach aimed to maximize advantages and minimize disadvantages of each strategy, completing gaps of scaffolds and ensuring the best assembly of *H*. *vastatrix* genome. The CLC HGAP strategy was employed to take advantage of the PacBio long read data to produce long contigs (Fig 1, blue boxes). The 17 longest read SMRT cells (P5-C3 chemistry) were used for this approach, as computational limitations prevented all 32 from being used. This approach produced a significant number of long contigs, however they were error prone (particularly short insert/deletion) and did not cover the complete genome.

SOAP De novo2 assembler was used for an Illumina-only de novo assembly (Fig 1, green boxes). PBJelly on 32 SMRT cells of long read sequencing was used to fill scaffolds and join contigs. This assembly had the longest metrics in terms of total contig/scaffold size, with scaffold size closely reflecting the expected genome size, although a significant amount of total data was in very short contigs. This assembly also did not validate well as it produced fewer annotated gene calls than other assemblies, a higher number of missing (20.2%) and fragmented (19.8%) conserved single-copy genes (BUSCO). Besides, reference mapping of the Illumina data back to the contigs resulted in higher rates of broken read pairs (61.9%) and a low rate of reference coverage (33% of the reference did not align reads) relative to other strategies.

The best results, were achieved by a hybrid *de novo* genome assembly approach (Fig 1, red boxes). Contigs that were produced using the HGAP assembly of PacBio long-read data (Fig 1, blue boxes) were leveraged as guide reads for a subsequent assembly of Illumina short-reads using the CLC Genomic Workbench. These resulting scaffolds were further filled and joined using PBJelly software, and a final error polishing step mapping Illumina reads back to the improved assembly. This strategy resulted in a final assembled genome of 549 Mbp in 118,162 scaffolds (< 0.5% gaps) (Table 1). Reference mapping of the approximately 260 million Illumina reads (130M read pairs) back to this genome assembly result in a 95.3% mapping rate. Interestingly, mapping of the same reads to only the contigs >2000 bp resulted in a very comparable mapping rate of 94.4%.

**Table 1:** Genomic features for the *Hemileia vastatrix* assembled genome (race XXXIII).

Genome Feature	Number
Total length	549,560,213 bp*
Number of scaffolds	118,162
Longest scaffold	137,364 bp
Shortest scaffold	143 bp
N50	9,965 bp
L50	16,308
Count (% length) scaffolds > 1Kb	92,276 (96.0%)
Count (% length) scaffolds > 2Kb	58,535 (87.5%)
Count (% length) scaffolds > 10Kb	16,236 (49.9%)
Count (% length) scaffolds > 50Kb	86 (1.0%)
A+T	66%
G+C	33.6%
N	0.44%

*bp—base pairs

### Genome annotation and gene prediction

Gene model predictions and gene product annotations were performed on the Hv33 genome assembly. Repetitive elements were identified in 118,162 scaffolds using the Repeat Masker software [51]. This analysis revealed that 82% of scaffolds contained repetitive elements, with transposable elements accounting for 43.6% of the total. Gypsy was found to be the most represented repetitive element in this genome (Table 2).

**Table 2:** Structural annotation analysis of *Hemileia vastatrix* genome (race XXXIII).

Type of repetitive element	Number of repetitive elements
**Retrotransposons**	
*Gypsy*	41,269
*Copy*	7,054
*LINEs*	426
*DIRs*	81
**Transposons**	
*EnSpm*	2,971
*Harbinger*	2,954
*Mariner*	837
*Helitron*	181
*MuDR*	16
*Hat*	10
***Others***	
Simple repeats	39,808
Low complexity	32,461

Annotation of the genome with Maker using custom-trained Augustus and SNAP gene models identified 33,483 predicted genes in the *H*. *vastatrix* genome with 33,153 putative protein coding genes supported by transcriptomic and/or genomic evidence from existing genome annotations within the order Pucciniales. Of these predicted genes, 13,364 protein coding genes could be supported by the currently available *H*. *vastatrix* transcriptomic data available with a Maker AED score less than 1. The. CEGMA analysis found that 96.4% of expected core genes were found in the genome, while BUSCO identified 91.7% (13.1% fragmented) of expected single copy genes (from 1335 conserved Basidiomycota proteins) in the protein annotations (S1 Fig). Of the expected BUSCO single-copy genes, 3.7% were duplicated which is in line with or lower than four existing reference genome assemblies from other species in the order Pucciniales. Additionally, reference mapping of our Illumina sequence reads back to the Hv33 genome result in 95.3% mapping rate, indicating that a significant fraction of the genomic sequence space is included in the Hv33 assembly.

### Functional annotation of proteome

Putative protein coding genes with transcriptomic support were compared against fungal protein sequences in the GenBank nr and UniProt databases. Results showed that 1,568 sequences were likely unique to *H*. *vastatrix* as these sequences did not have sequence similarity to any other sequence in the queried databases. Of sequences with a blast hit (*e*-value < 10^−5^), 72.2% were most similar to *Puccinia graminis*, 27.0% were more similar to *Melampsora larici-populina*, and 0.8% were most similar to sequences from *H*. *vastatrix* that had already been deposited in the databases.

A functional clustering analysis was performed by using the Blast2GO program for 11,466 protein sequences found to be significantly similar (e-value < 10^−5^) to those obtained from GenBank and Uniprot databases. From these sequences, 7,139 were further computationally characterized. A Gene Ontology hierarchical analysis revealed 12,501 annotation clusters in categories of molecular function, biological process, and cellular component (some proteins were annotated in two or more categories). The largest number of annotated genes (6,331) had a molecular function, with ‘ion binding’ and ‘hydrolase activity’ being the most represented GO categories with 889 and 538 proteins, respectively (S2A Fig). A total of 3,887 annotated genes had biological processes, followed by 2,283 annotated genes with a cellular component assigned. For the biological process category, ‘cellular metabolic process’, ‘primary metabolic process’ and ‘organic substance metabolic process’ were more represented with 1,243, 1,086 and 1,086 protein sequences, respectively (S2B Fig). For the cellular component category, the following GO-terms were most represented: ‘cell part’, ‘membrane-bounded organelle’, ‘protein complex’ and ‘non-membrane-bounded organelle', comprising 1,326, 669, 511, and 464 proteins, respectively (S2C Fig). The analysis performed for the hierarchical levels numbers 6 and 8, led us to confirm that as these proteins are essentially associated with the nucleus, cytosol, ribosome, mitochondria, Golgi apparatus, and endoplasmic reticulum (S2D Fig).

### Prediction and functional annotation of secretome

To identify potentially secreted proteins, we applied a sequential bioinformatics pipeline that employed SignalP, TargetP, and TMHMM network-based tools to analyze the predicted proteins with transcriptome support. Results revealed 615 putative secreted proteins. But those numbers drop to 452 after redundancy analysis. This subset of proteins was significantly similar (*e*-value < 10^−5^) mainly to proteins from *P*. *graminis* f. sp. *tritici* (385) and *M*. *larici-populina* (109). In addition, 39 protein sequences showed similarity with previously deposited sequences from *H*. *vastatrix*, and 72 returned no results, indicating their uniqueness to the *H*. *vastatrix* race XXXIII genome.

**Fig 2:**
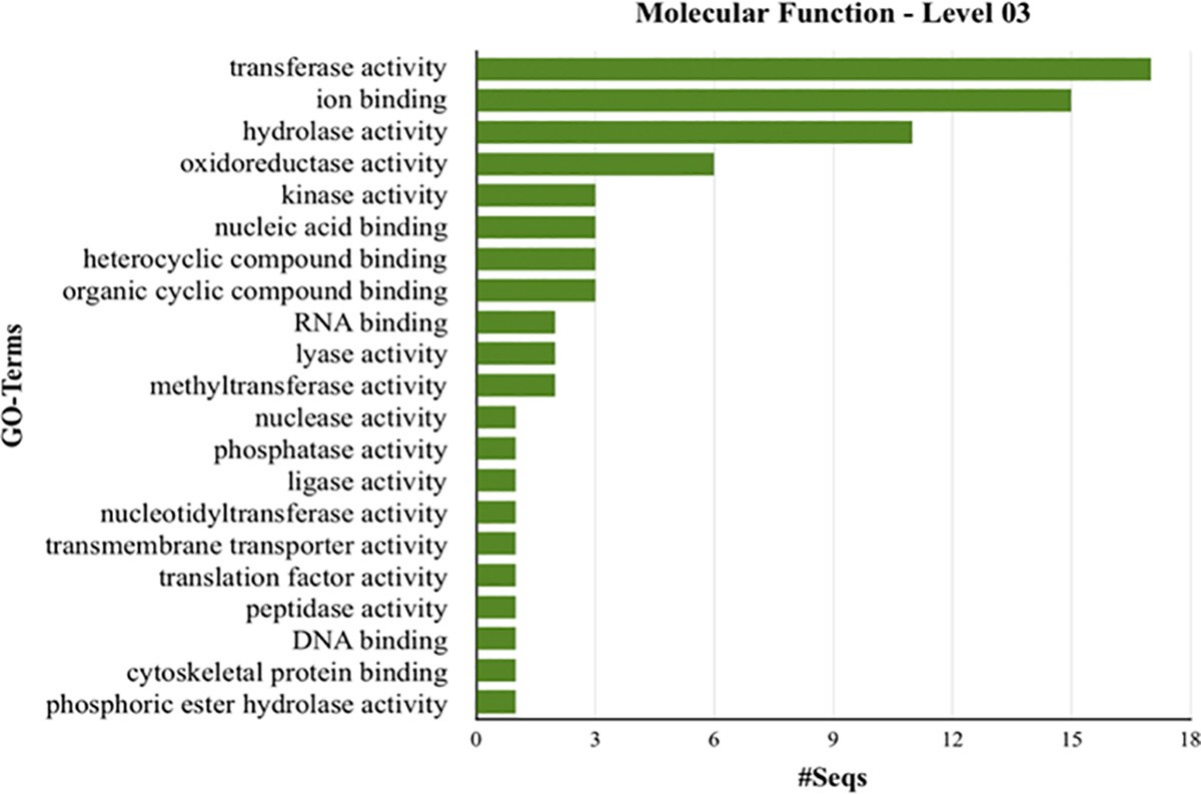
Functional clustering analysis of secreted proteins identified in *H*. *vastatrix* genome, race XXXIII. Secreted proteins were found to be significantly similar (*e*-value < 10^−5^) to protein sequences obtained from NCBI and UNIPROT databases by using the Blast2GO bioinformatics platform. The y-axis consists of GO-terms described in the molecular function category for the hierarchical level #3 and the x-axis consists of protein sequences found for each GO-term in this category.

The Blast2GO program was used for functional clustering of 543 proteins found to be similar to sequences of proteins obtained from the NCBI and UNIPROT databases. From these proteins, 161 were clustered in categories of molecular function. The largest number of proteins was associated with transferase activity, ion binding and hydrolase activity (Fig 2). By Wolf Psort analyses (Table 3), most of the secreted proteins had subcellular location assigned as mitochondria (241) and extracellular space (186).

**Table 3:** Prediction of subcellular localization of *Hemileia vastatrix* (race XXXIII) secretome using Wolf Psort.

Subcellular localization prediction	Number of proteins	Percentage (%)
Cytosol	26	4.23
Endoplasmic reticulum	3	0.49
Extracellular space	186	30.24
Golgi	1	0.16
Mitochondria	241	39.19
Nucleus	88	14.31
Peroxisome	37	6.02
Plasma membrane	26	4.23
No hit	7	1.14

**Fig 3:**
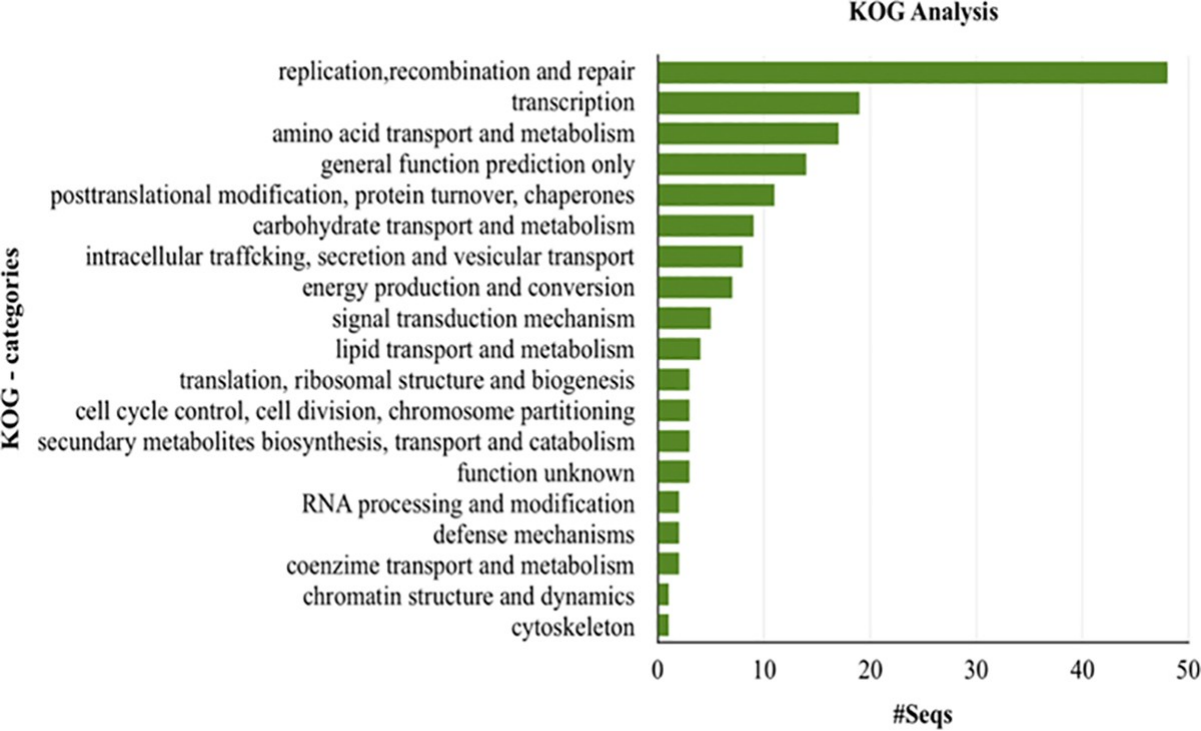
Functional clustering analysis of secreted proteins found in the genome of *H*. *vastatrix* race XXXIII, which specific signatures in KOG categories. The y-axis consists of KOG categories, and the x-axis consists of protein sequences found for each KOG category.

The KOG category analysis showed that approximately 27% of 615 secreted proteins have specific signatures and the most represented being replication, recombination and repair, transcription, and amino acid transport and metabolism (Fig 3).

We further analyzed whether the putative secreted proteins of *H*. *vastatrix* race XXXIII contained other effector-like sequences for the presence of cysteine residues, which are indicative signs of secretion in other plant pathogens. Cysteine residues were found in approximately 93% of the putative secreted proteins, and the majority contained either two, three, or four residues in their sequences. In addition, the amino acid motifs commonly found in fungal effectors, [YWF]xC, CxxC and CxxxC, were found in 365 of the secreted protein, either alone or grouped.

### Transcriptional analysis of potential effector proteins

**Fig 4:**
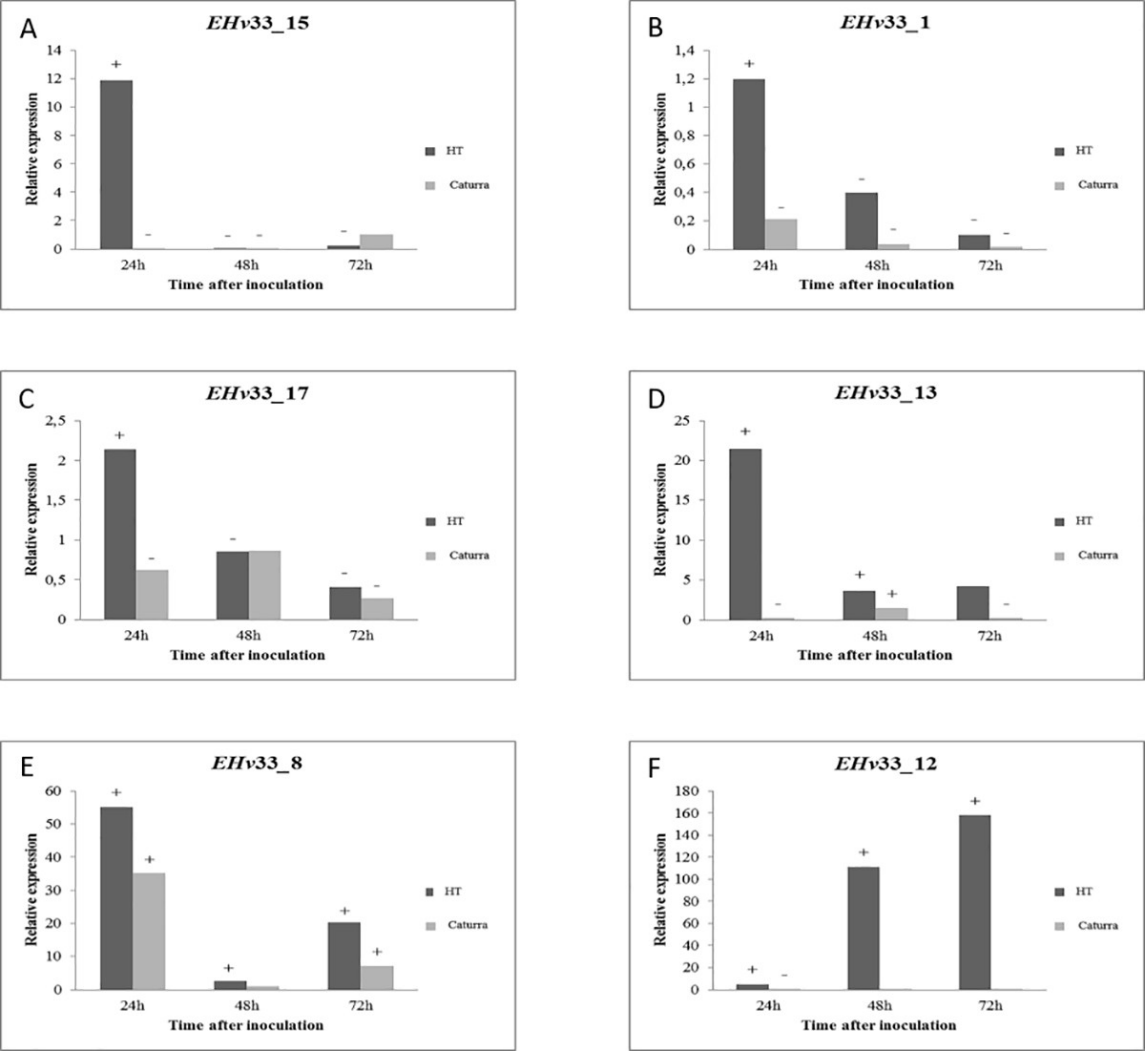
Quantitative RT-PCR-based analysis of gene expression performed for six EHv33 genes that encode putative candidate secreted effector proteins of *H*. *vastatrix*, race XXXIII. The relative expression pattern of target genes was estimated in plant samples of the hybrid of Timor and Caturra. Data were recorded at 12, 24, 48 and 72 hours after inoculation. The period of 12 hours after inoculation was used as reference sample. The expression level of target genes was normalized by using two endogenous genes of *H*. *vastatrix*, namely, β-tubulin and CytIII. (A) EHv33_15: the highest level of gene expression was recorded at 24 hours after inoculation (hai). (B) EHv33_1: the highest level of gene expression was recorded at 24 hai and, then, decreased over time. (C) EHv33_17: the highest level of gene expression was recorded at 24 hai and, then, decreased over time. (D) EHv33_13: the highest level of gene expression was recorded at 24 hai and, then, decreased at 48 hai and, at the end, the expression estimate kept constant at 72 hai. (E) EHv33_8: the highest level of gene expression was recorded at 24 hai and, then, decreased at 48 hai and increased again at 72 hai. (F) EHv33_12: the increase of gene expression was recorded at 48 hai but, the highest level of gene expression was only recorded at 72 hai.

**Fig 5:**
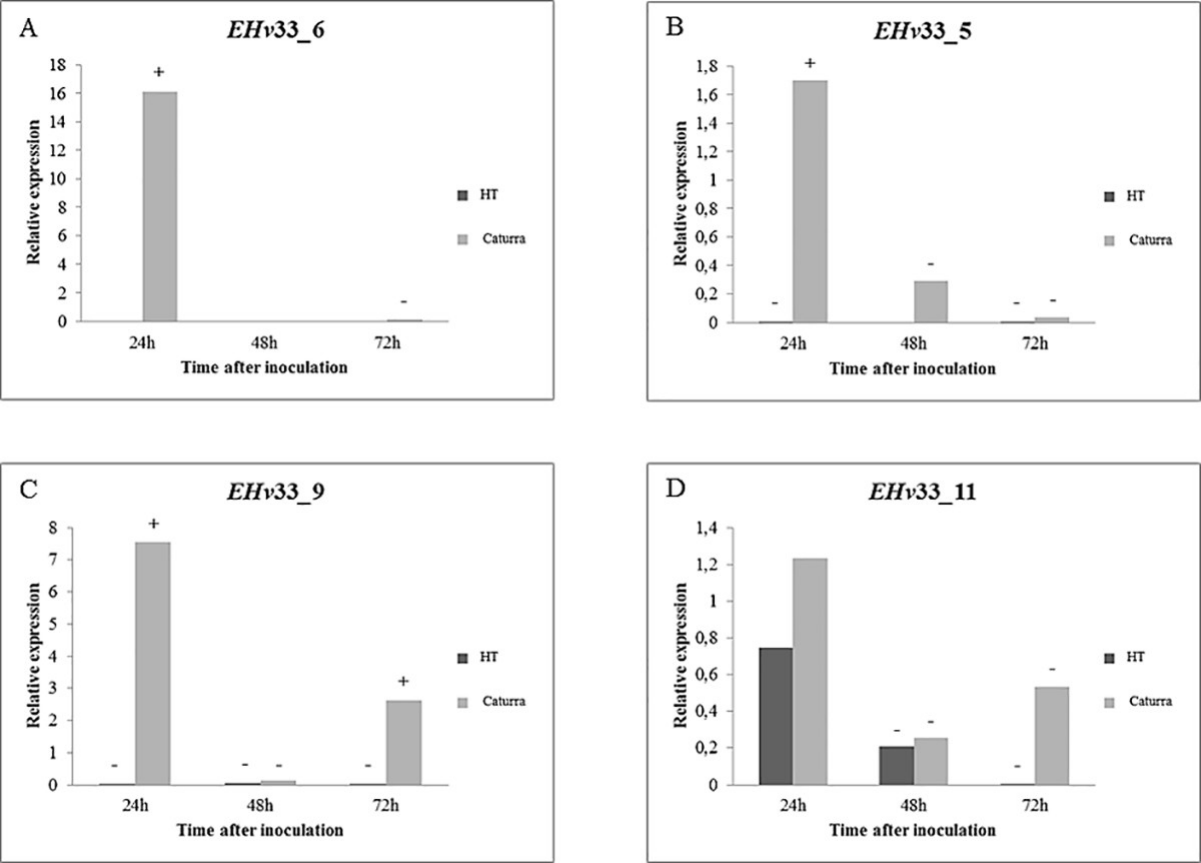
Quantitative RT-PCR-based analysis of gene expression performed for four EHv33 genes that encode putative candidate secreted effector proteins of *H*. *vastatrix*, race XXXIII. The relative expression pattern of target genes was estimated in plant samples of the hybrid of Timor and Caturra. Data were recorded at 12, 24, 48 and 72 hours after inoculation. The period of 12 hours after inoculation was used as reference sample. The expression level of target genes was normalized by using two endogenous genes of *H*. *vastatrix*, namely, β-tubulin and CytIII. (A) EHv33_6: the highest level of gene expression was recorded at 24 hours after inoculation (hai). (B) EHv33_5: the highest level of gene expression was recorded at 24 hai and, then, decreased over time. (C) EHv33_9: the highest level of gene expression was recorded at 24 hai and, then, decreased at 48 hai, and increased again at 72 hai. (D) EHv33_11: the highest level of gene expression was recorded at 24 hai and, then, decreased at 48 hai, and increased again at 72 hai.

**Fig 6:**
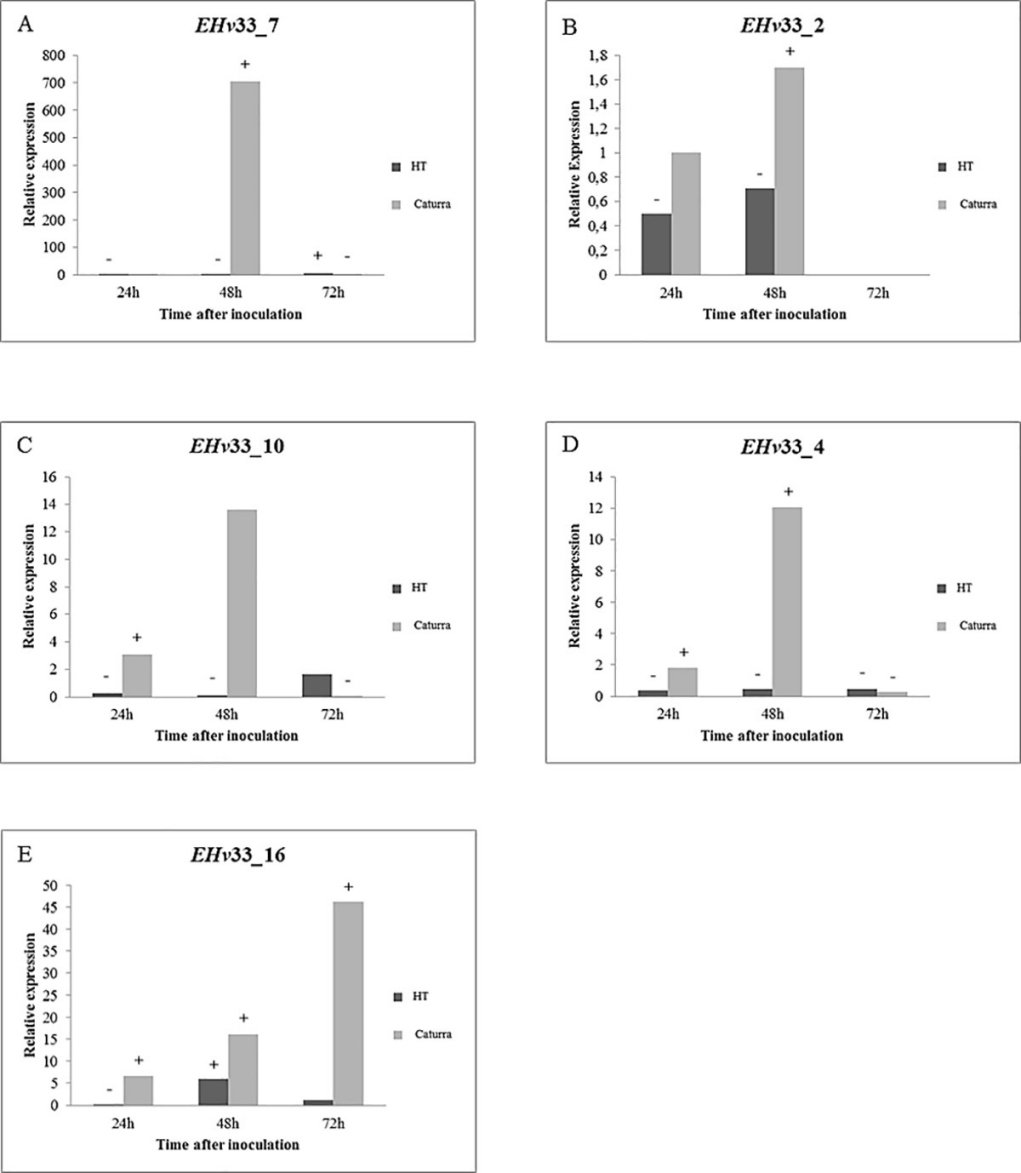
Quantitative RT-PCR-based analysis of gene expression performed for five EHv33 genes that encode putative candidate secreted effector proteins of *H*. *vastatrix*, race XXXIII. The relative expression pattern of target genes was estimated in plant samples of the hybrid of Timor and Caturra. Data were recorded at 12, 24, 48 and 72 hours after inoculation. The period of 12 hours after inoculation was used as reference sample. The expression level of target genes was normalized by using two endogenous genes of *H*. *vastatrix*, namely, β-tubulin and CytIII. (A) EHv33_7: the highest level of gene expression was recorded at 48 hours after inoculation (hai). (B) EHv33_2: the highest level of gene expression was recorded at 48 hai and, then, decreased over time. (C) EHv33_10: the highest level of gene expression was recorded at 48 hai and, then, decreased over time. (D) EHv33_4: the highest level of gene expression was recorded at 48 hai and, then, decreased over time. (E) EHv33_16: the highest level of gene expression was recorded at 72 hai.

Seventeen genes that encode potential effector proteins identified in this study were selected for gene expression analysis using real-time quantitative RT-PCR (Figs 4–6). Some of these selected genes did not match with any other sequence in the queried databases (see methods for details) and other genes were identified as secreted protein of *H*. *vastatrix*, thus suggesting they are likely unique to *H*. *vastatrix*. These 17 potential effector proteins have predicted secretion signal and cysteine residues in their amino acid sequences and were also selected by BLAST hits to the database of *H*. *vastatrix* secretome from previously published studies [36, 48] and from a database of RNA-Seq libraries of resistant coffee infected with *H*. *vastatrix* (S2 Table).

Six of these genes were significantly expressed in the incompatible interaction (Fig 4) and nine were expressed in the compatible interaction (Fig 5 and Fig 6). Two genes, EHv33_3 and EHv33_14, did not show significant differences between compatible or incompatible interactions (S3 Fig).

**Fig 7:**
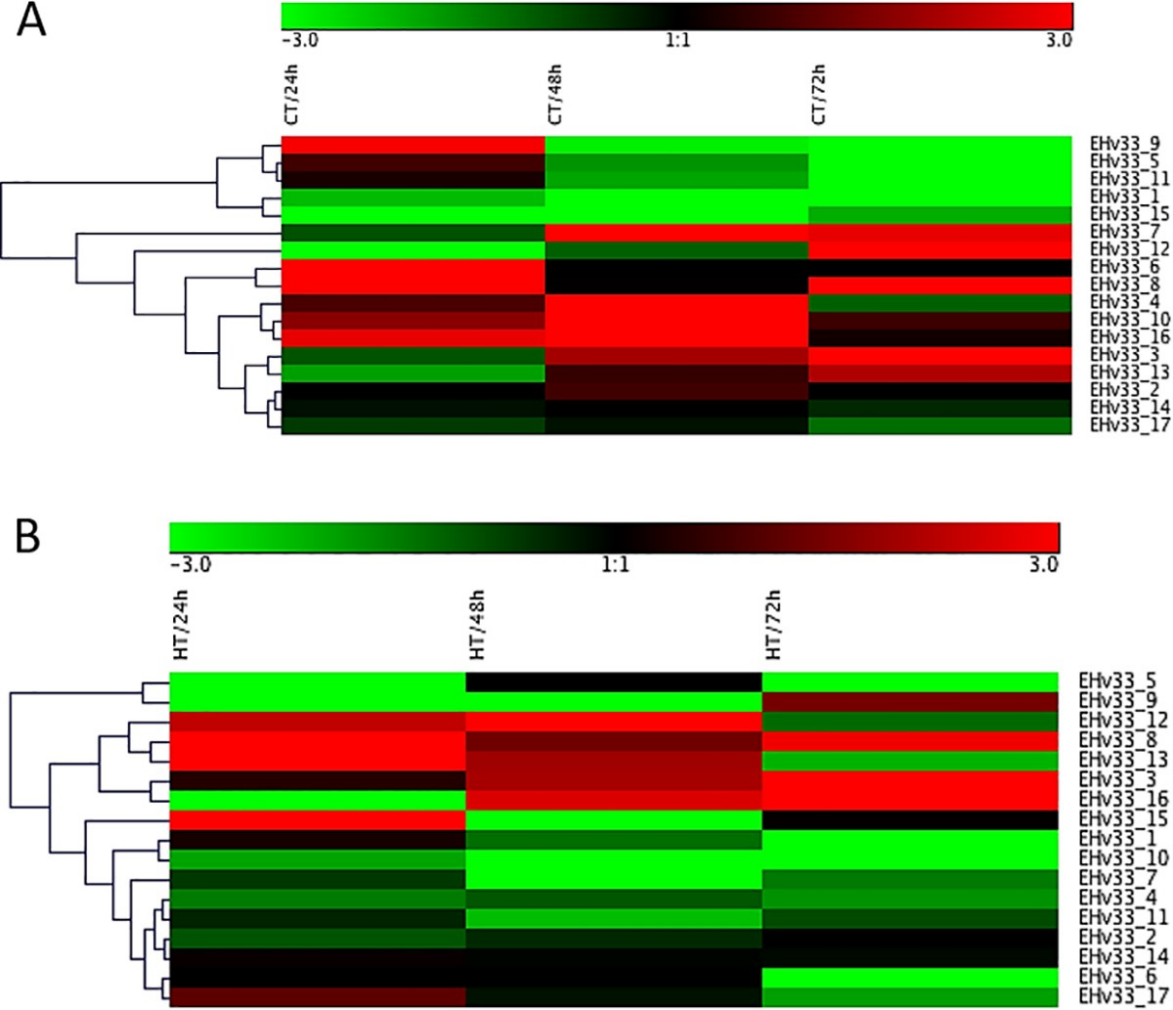
Heat map of 17 genes expression profile obtained from quantitative real time PCR. (A): genes expression profile (17) in resistant plant (Híbrido de Timor) during 24, 48 and 72 hai. (B): genes expression profile (17) in susceptible plant (Caturra) during 24, 48 and 72 hai. Red indicates a relatively high level of up-regulated, whereas green indicates a relatively low level of up-regulation.

A peak in expression was observed at 24 hours after inoculation (hai) in the incompatible interaction (no disease) for the following putative effector genes: EHv33_15, EHv33_1, EHv33_17, EHv33_13 and EHv33_8 (Fig 4A–4E). The EHv33_12 gene showed an expression increase at 48 hai and, the highest expression level of this gene was at approximately 72 hai (Fig 4F). The remaining EHv33 genes showed higher expression levels in the compatible interaction (Figs 5 and 7), where disease is the outcome. Genes EHv33_6, EHv33_5, EHv33_9 and EHv33_11 (Fig 5A–5D) showed a peak in expression at 24 hai; and EHv33_7, EHv33_2, EHv33_10 and EHv33_4 (Fig 6A–6D) at 48 hai, followed by a decrease in expression at 72 hai. The EHv33_16 gene showed significant expression level at 24 hai, followed by a gradual increase over time. The highest transcript accumulation was recorded at 72 hai (Fig 6E). A summary of these data is showed as a heat map (Fig 7) in susceptible (Caturra, Fig 7A) and resistant plants (Híbrido de Timor, Fig 7B). The heat map was generated by a log transformation of the RT-qPCR data presented as ^ΔΔ^CT.

## Discussion

### *H*. *vastatrix* race XXXIII genome sequence summary

We utilized PacBio RS II and Illumina HiSeq to sequence the genome of the coffee rust fungus, *H*. *vastatrix* race XXXIII. This hybrid assembly approach of long and short reads yielded a genome approximately 576 Mb in size. We have noted that the population of contigs shorter than 2 kb appear to be dominated by fragments of regions found elsewhere in the assembly, but with enough sequence heterogeneity to exclude them from the longer contigs and to justify retaining them in this assembly. While such repetitive sequence data may represent allelic diversity, an alternative hypothesis is that this genome has undergone fairly large-scale duplication events. Such events could not only contribute to the difficulties that have arisen in assembling this genome, but could also explain how > 95% of sequence reads can recruit to a genome assembly that is 200-300Mbp shorter than the experimentally-predicted genome size of 733.5 Mb [24] to 796.8 Mb [25].

The combined use of a single monopustular isolate and long read sequencing (PacBio) in our approach was fundamental for obtaining this assembled genome with a higher level of contiguity (N50 = ~10.0 kb; Table 1), than the previous assembly of HvCat genome (N50 = ~1.6 kb; total genome 330 Mbp [23]). While the Hv33 genome assembly remains fragmented, it exhibited increased genome coverage, contiguity, and performs better for reference mapping of high-throughput sequencing data than the previous assembly. Reference mapping of the underlying sequence data from the HvCat assembly to Hv33 resulted in 80.8% of reads mapping, while a reciprocal analysis mapping our raw data to the HvCat genome resulted in a mapping rate of 60.4%. Other indicators including the >95% rate of raw data mapping to the Hv33 genome and the BUSCO results indicating that >90% of conserved single copy proteins are present, with a rate of duplication in line with other rust genomes (S1 Fig), also point to an assembly that covers the majority of the genomic sequence space.

*H*. *vastatrix* genome has been estimated to be one of the largest fungal genomes, only smaller than those predicted based on flow cytometry for *Gymnosporangium confusum* (893.2 Mb) and *Puccinia chrysanthemi* (806.5 Mb) [25], however these genomes have not yet been sequenced. Other economically important rusts, such as *P*. *graminis* f.sp. *tritici* (88.6 Mb), *P*. *striiformis* f.sp. *tritici* (64.8 Mb), *M*. *larici-populina* (101,1Mb), and *M*. *lini* (189 Mb), have relatively smaller genomes [19] [21] [22]. Parameters such as lifestyle, intraspecific genetic variability, selection pressure, and genetic drift, are considered determining factors for fungal genome size [52] [53].

Having large genomes does not mean having more genes, as genome expansion may occur not only by acquisition of new genes, but also by increasing repetitive regions or by transposable element proliferation during the evolutionary process [52]. Our study identified about 82% of scaffolds contained repetitive elements, from which 43.6% consisted of transposable elements (Table 2). Studies involving different species responsible for leaf rusts, as *P*. *striiformis* f.sp. *tritici*, *P*. *graminis* f.sp. *tritici*, *M*. *larici-populina*, *M*. *lini* and *H*. *vastatrix*, showed that 17.8%, 42.5%, 40.5%, 31.67% and 48% of repetitive elements, respectively, consist of transposable elements [19] [21] [23] [22]. Differences in terms of transposable elements content may occur due to a very large genome size, which can contain large proportion of repetitive regions; and methods for hybrid genome assembly used, which may overestimate the real number of repetitive regions. The transposable elements are important sources of variability that occurs by means of mutations during the evolutionary process. The short lifespan of plant pathogenic organisms, however, makes them reproduce more often than their hosts, consequently evolving faster. Because of this, it is suggested that *H*. *vastatrix* has a great capacity of adaptation and high genetic variability during its co-evolution with the host over time.

The high similarity (~72%) between predicted proteins in *H*. *vastatrix* genome and protein sequences of *P*. *graminis* f.sp. *tritici* may not be solely because they belong to the same order, but also because they may have a similar gene content necessary for pathogenicity and may employ a similar pathogenic process. An advantageous point is that the genome sequence of *P*. *graminis* f.sp. *tritici* is publicly available [19] and can be used for further detailed comparative analysis of both pathogens.

The discovery of species-specific proteins and effectors is not trivial; while searches typically utilize criteria like being small and cysteine-rich to identify candidates, not all will function as effectors, and not all effectors will possess these hallmarks [54]. In the *H*. *vastatrix* genome, 12% of predicted proteins have no significant similarity (no matches) to protein sequences of fungi obtained from the NCBI and UNIPROT databases, and thus were considered unique to *H*. *vastatrix*.

### Candidate secreted effector protein (CSEPs) of *H*. *vastatrix*

We analyzed predicted proteins of *H*. *vastatrix* genome, and identified 615 candidate secreted effector proteins (CSEPs). After redundancy analysis, this number dropped to 452 CSEPs (S3 Table). Previous studies predicted various numbers of CSEPs for *H*. *vastatrix*, as being 382 [36], 516 [48], from 659 to 775 [23] and 146 [55]. This variation in number of CSEPs is due to the type of data, software, and approaches used in each analysis. For instance, the range of predicted CSEPs, from 659 to 775, observed by [23], is indeed dependent on the prediction program used (659 CSEPs predicted using PProwler and 775 CSEPs using SignalP) and did not considered the analysis by TargetP (for subcellular localization of proteins) and TMHMM (to discriminate soluble and membrane proteins), which would decrease the predicted number. The smaller number of CSEPs, 382 [36], 516 [48], and 146 [55] was based on the analysis of expressed sequence tags (ESTs), thus, limiting identification of the full set of CSEPs. Using BLASTp, none of our 615 CSEPs were found in the study by [55]. However, we had 68 and 84 CSEPs found in studies by the [36] and [48].

Interestingly, when comparing *H*. *vastatrix* CSEPs with others rust fungi, 62.7% (385) of the *H*. *vastatrix* CSEPs are similar to *P*. *graminis* f. sp. *tritici* proteins, whereas only 17.7% (109) are similar to *M*. *larici-populina* proteins.

The classical secretory pathway [56] delivers the majority of effector proteins of the plant pathogenic fungi described thus far. Sequences of effector proteins frequently have no obvious similarity to each other or to other proteins in current databases [57], i.e., they are found to be species-specific effectors. Only 39 sequences from the predicted secretome had similarity to the public sequences of *H*. *vastatrix*. This low similarity can be explained by the small number of proteins of *H*. *vastatrix* available in the GenBank to date. In addition, 72 secreted proteins did not match to any protein sequence obtained from the databases. These proteins potentially represent novel genes and were considered unique to *H*. *vastatrix*, as they have not yet been described. The 111 (39+72) CSEPs of *H*. *vastatrix* were annotated using the Pfam software and no function has been assigned to them. Thus, the majority of EHv33 sequences reported in this study have no conserved domain described by the Pfam domain.

About 93% of 615 secreted proteins of *H*. *vastatrix* showed cysteine residues, the majority consisting of two, three or four residues in the sequence. According to Stergiopolous and de Wit [58], fungal effector proteins are usually rich in cysteine residues. These residues confer stability to proteins in extracellular space by means of formation of intra-molecular disulfide bridges that are characterized by their importance for structure and function. Changes in amino acid sequence may occur without changing the topology of proteins, except if it occurs in cysteine residues, making it ideal for the recognition and specificity [59]. Thus, the greater number of cysteine residues in an effector protein, the faster is its capacity for changes under any environmental selection pressure, thus favoring the evolution of new effectors and even new fungal physiological races. We identified large (>150 aa) proteins with up to 16 cysteine residues. While these proteins might not be considered typical effectors due to their size, the number of cysteine residues could make them candidate effectors with a high capacity for evolution [60].

Bioinformatics analysis can be used to identify motifs responsible for effector trafficking into host cells by predicting the presence and location of signal peptides [17]. Motifs of effectors such as [YWF]xC, CxxC and CxxxC have been found in conserved families of pathogen effectors belonging to the Order Pucciniales [19]. In this study, these latter domains were found in 365 secreted proteins. While this indicates that these proteins may function as effectors, functional characterization assays are needed to decipher the molecular role of these proteins in pathogenesis and/or on pathogen fitness.

### Temporal expression of *H*. *vastatrix* race XXXIII CSEPs in diverging host compatibility

Global expression of the predicted candidate effectors may indicate the effectors that are crucial for the disease establishment and progression, especially, the candidate effectors highly expressed during early infection [52]. We analyzed relative expression of 17 *H*. *vastatrix* race XXXIII CSEPs during a time course interaction with a susceptible and a resistant genotype, and our results show differing results depending on the cultivar used. These 17 candidates were chosen based upon possessing a predicted signal sequence and cysteine residues, and their uniqueness to *H*. *vastatrix*. More CSEPs were highly expressed in the compatible interaction than the incompatible interaction, which is expected as the effectors play a role in manipulating host defense and contribute to disease development. Six effectors showed expression during the incompatible interaction with Híbrido de Timor, the majority showing expression peaks at 24 hours after inoculation followed by a decrease. It is interesting to speculate that these effectors might play a role in defense and recognition by cognate R genes in the plant, however further experimentation is required to fully understand their functions.

A previous study from our group [35] showed that the interaction between a resistant genotype and race XXXIII starts very early, even before fungal penetration. Microscopic observations in the pathosystem Híbrido de Timor and *H*. *vastatrix* race XXXII revealed that cytological responses induced by the fungus can be observed in stomatal cells by 17 hours after infection (hai) and corresponded to hypersensitive-like cell response. Therefore, the fungus ceased its growth in the early stages of infection process, frequently in the penetration hyphae stage, indicating a pre-haustorial resistance. This resistance response was observed in 18% of infection sites at 17hai, reaching 65% and 93% at 24hai and 96hai, respectively [35]. Many effectors secreted by germinating spores are involved in signal transduction and establishment of the infection processes, in an attempt to suppress PAMP-triggered immunity (PTI), and then are recognized by plant receptors in resistant plants, triggering defense responses (effector-triggered immunity or ETI) [36] [61] [48]. This type of plant resistance, known as pre-haustorial resistance, was detected in coffee-rust interaction previously [62] [14]. From our predicted secretome, we found six candidates effectors significantly expressed only in the incompatible interaction and of these, five, EHv33_15, EHv33_1, EHv33_17, EHv33_13 and EHv33_8, were more abundant at 24 hours. This result suggests that they are pre-haustorial candidate effectors that might be attempting to suppress PTI, but additional experiments are required to confirm this further.

Diniz et al [63] found similar result to our prior cytological observations and suggested that this rapid plant defense response, which prevents haustorium formation, may be the basis for durable resistance to races of this fungal species. Therefore, those five candidate effectors identified can play important function in coffee resistance to the rust pathogen. On the other hand, if the pathogen overcomes the pre-haustorial resistance, the resistant plants will likely delay fungal growth in early stages of infection. This strategy is known as post-haustorial resistance [64] [10] and is usually shown after the formation of primary haustorium, which occurs about 48 hours after inoculation of *H*. *vastatrix* on coffee leaves. In our study, the post-haustorial expression pattern was detected for EHv33_12 effector candidate only, may be an ineffective attempt to suppress ETI.

*H*. *vastatrix* differentiates several specialized infection structures in the infection process. In susceptible genotypes, after the primary haustorium formation, the fungal growth continues, resulting in the formation of many intercellular hyphae and haustoria in mesophyll cells. Those changes in fungal development occur from 48 to 72 hours after inoculation [62] [48]. In this study, some effector candidates showed high expression levels in susceptible genotypes from 48 to 72 hours after inoculation. Thus, we can infer that the following genes are effector candidates possibly translocated into host cells via haustorium: EHv33_7, EHv33_2, EHv33_10, EHv33_4 and EHv33_16.

Overall, all these identified genes, pre- and post-haustorial candidate effectors, can be exploited to assist breeding programs, as biotechnological tools (effectoromics). Effectors are emerging as tools to accelerate and improve the identification and functional characterization of resistance genes that encode plant proteins able to recognize these effectors [18]. Thus, they can be used as markers to identify resistant genotypes in coffee breeding programs.

### Ongoing challenges and future directions on *H*. *vastatrix* research

The genome sequence of the fungus *H*. *vastatrix* presented here is intended to help advance the knowledge of the pathogen and how it interacts with the plant successfully. With the more accurate number of predicted proteins and effectors, analyses for a more detailed understanding of their functional role and the set of main players in the interaction can now be performed. All the new findings can be useful in the development of molecular markers to distinguish races and, thus, provide a helpful tool to outperform difficulties found in the use of differential coffee cultivars.

## Supporting information

S1 Fig Assessment of the completeness of assembly/annotation with comparison to other genome assemblies from the order Puccinales by using the BUSCO bioinformatics platform. HvCat refers to *H*. *vastatrix* isolate (GCA_003057935.1); Mp: *Melampsora larici-populina* 98AG31 (GCF_000204055.1); Ml: *Melampsora lini* CH5 (JGI Genome portal: https://genome.jgi.doe.gov/Melli1/Melli1.info.html); Pg: *Puccinia graminis* f. sp. *tritici* CRL 75-36-700-3 (GCF_000149925.1); and Ps: *Puccinia striiformis* f. sp. *tritici* (GCA_001936605.2).
(TIF)

S2 Fig Functional clustering analysis of proteins identified in *H. vastatrix* genome, race XXXIII, which were found to be significantly similar (e-value < 10−5) to protein sequences obtained from NCBI and UNIPROT databases by using the Blast2GO bioinformatics platform. **A)** The y-axis consists of GO-terms described in the molecular function category for the hierarchical level #3. The x-axis consists of protein sequences found for each GO-term in this category. **B)** The y-axis consists of GO-terms described in the biological process category for the hierarchical level #3. The x-axis consists of protein sequences found for each GO-term in this category. **C)** The y-axis consists of GO-terms described in the cellular component category for the hierarchical level #3. The x-axis consists of protein sequences found for each GO-term in this category. **D)** The y-axis consists of GO-terms described in the cellular component category for the hierarchical levels #6 and #8. The x-axis consists of protein sequences found for each GO-term in this category.
(TIF)

S3 Fig Quantitative RT-PCR-based analysis of gene expression performed for two EHv33 genes that encode putative candidate secreted effector proteins of *H. vastatrix*, race XXXIII. The relative expression pattern of target genes was estimated in plant samples of the hybrid of Timor and Caturra. Data were recorded at 12, 24, 48 and 72 hours after inoculation. The period of 12 hours after inoculation was used as reference sample. The expression level of target genes was normalized by using two endogenous genes of *H*. *vastatrix*, namely, β-tubulin and CytIII. **A)** EHv33_3: the gene expression increased over time, and the highest level was recorded at 72 hours after inoculation either in compatible or incompatible interaction. **B)** EHv33_14: there is no significant expression difference over time either in compatible or incompatible interaction.
(TIF)

S1 Table Primers used for RT-PCR and qRT-PCR, with sequences designed using the Primer Express 2.0 software. a correspond to β-tubulin; b: cytochrome c oxidase subunit III; c: glyceraldehyde-3-phosphate dehydrogenase; EHv33: *Hemileia vastatrix* candidate effectors genes race XXXIII.
(XLSX)

S2 Table Selection of 17 genes that encode potential effector proteins of *Hemileia vastatrix* race XXXIII identified in this study for gene expression analysis by RT-qPCR. *Data Base = NCBI/UNIPROT, RNA-Seq library by Lopes 2015 and Secreted proteins by Fernandez et al 2012 and Talhinhas et al 2014; ** No hits found = no hits found with any data base; ***Hits found = Hits found with NCBI/Uniprot data base with secreted protein *Hemileia vastatrix*.
(XLSX)

S3 Table Putative Secreted Proteins (Singlets) *Hemileia vastatrix* race XXXIII. (XLSX)
