# Supplementary figures and images for: Genome sequencing and transcript analysis of Hemileia vastatrix reveal expression dynamics of candidate effectors dependent on host compatibility
Source: PLoS One. 2019 Apr 18;14(4):e0215598. doi: 10.1371/journal.pone.0215598 (PMC6472787; doi:10.1371/journal.pone.0215598)

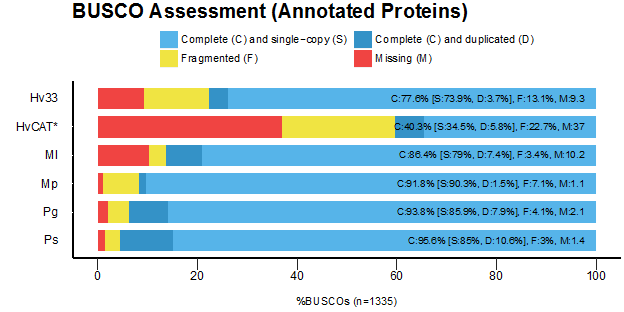

Supplement: S1 Fig — HvCat refers to H. vastatrix isolate (GCA_003057935.1); Mp: Melampsora larici-populina 98AG31 (GCF_000204055.1); Ml: Melampsora lini CH5 (JGI Genome portal: https://genome.jgi.doe.gov/Melli1/Melli1.info.html); Pg: Puccinia graminis f. sp. tritici CRL 75-36-700-3 (GCF_000149925.1); and Ps: Puccinia striiformis f. sp. tritici (GCA_001936605.2). (TIF) [file pone.0215598.s001.tif]

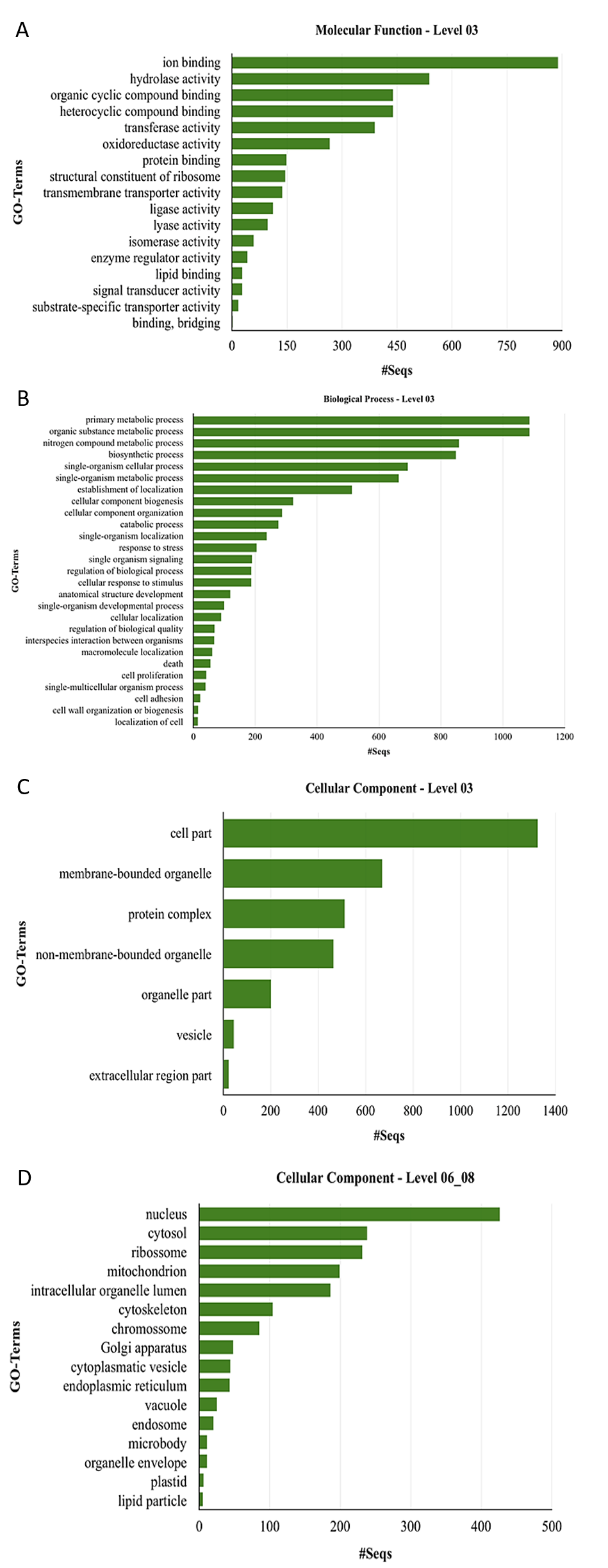

Supplement: S2 Fig — A) The y-axis consists of GO-terms described in the molecular function category for the hierarchical level #3. The x-axis consists of protein sequences found for each GO-term in this category. B) The y-axis consists of GO-terms described in the biological process category for the hierarchical level #3. The x-axis consists of protein sequences found for each GO-term in this category. C) The y-axis consists of GO-terms described in the cellular component category for the hierarchical level #3. The x-axis consists of protein sequences found for each GO-term in this category. D) The y-axis consists of GO-terms described in the cellular component category for the hierarchical levels #6 and #8. The x-axis consists of protein sequences found for each GO-term in this category. (TIF) [file pone.0215598.s002.tif]

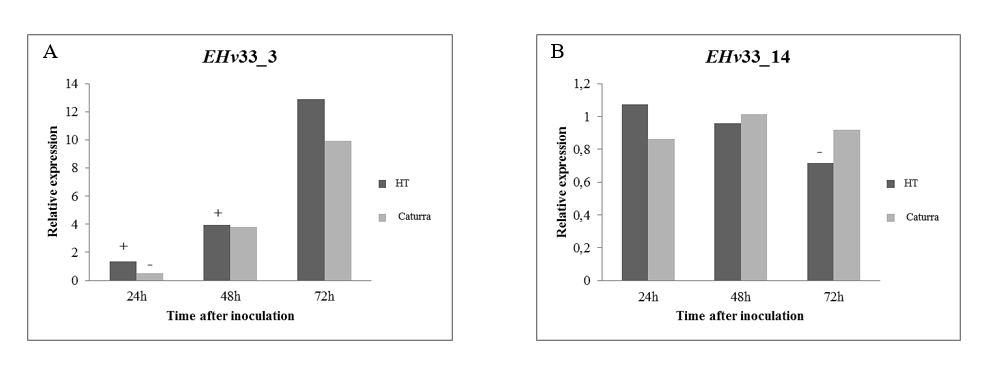

Supplement: S3 Fig — The relative expression pattern of target genes was estimated in plant samples of the hybrid of Timor and Caturra. Data were recorded at 12, 24, 48 and 72 hours after inoculation. The period of 12 hours after inoculation was used as reference sample. The expression level of target genes was normalized by using two endogenous genes of H. vastatrix, namely, β-tubulin and CytIII. A) EHv33_3: the gene expression increased over time, and the highest level was recorded at 72 hours after inoculation either in compatible or incompatible interaction. B) EHv33_14: there is no significant expression difference over time either in compatible or incompatible interaction. (TIF) [file pone.0215598.s003.tif]
